# Effect of Type and Dose of Exercise on Neuropathic Pain after Experimental Sciatic Nerve Injury: a Preclinical Systematic Review and Meta-analysis

**DOI:** 10.1016/j.jpain.2023.01.011

**Published:** 2023-01-21

**Authors:** Luis Matesanz-García, Clément Billerot, Joel Fundaun, Annina B. Schmid

**Affiliations:** a Nuffield Department of Clinical Neurosciences, University of Oxford, Oxford, United Kingdom; b Department of Physical Therapy, Occupational Therapy, Rehabilitation and Physical Medicine, Escuela Internacional de Doctorado, Universidad Rey Juan Carlos, Alcorcón, Spain; c Department of Physiotherapy, Centro Superior de Estudios Universitarios La Salle. Universidad Autónoma de Madrid, Madrid, Spain; d Faculty of biology. Euro-Mediterranean Master in Neurosciences and Biotechnology, Université de Bordeaux, Bordeaux, France

**Keywords:** Sciatic Nerve Injury, Neuropathic Pain, Exercise, Hyposensitivity, Biomarker

## Abstract

This preclinical systematic review aimed to determine the effectiveness of different types and doses of exercise on pain behaviour and biomarkers in preclinical models of focal neuropathic pain.

We searched MEDLINE, EMBASE, Web of Science, PubMed, SCOPUS, CINAHL and Cochrane library from inception to November 2022 for preclinical studies evaluating the effect of exercise compared to control interventions on neuropathic pain behaviour after experimental sciatic nerve injury. If possible, data were meta-analysed using random effect models with inverse-variance weighting.

Thirty-seven studies were included and 26 meta-analysed. Risk of bias (SYRCLE tool) remained unclear in most studies and reporting quality (CAMARADES) was variable. Exercise reduced mechanical (SMD 0.53 (95% CI 0.31, 0.74), p= 0.0001, I^2^=0%, n=364), heat (0.32 (0.07,0.57), p=0.01, I^2^=0%, n=266) and cold hypersensitivity (0.51 (0.03, 1.0), p=0.04, I^2^=0%, n=90) compared to control interventions. No relationship was apparent between exercise duration or intensity and antinociception. Exercise modulated biomarkers related to different systems (e.g., immune system, neurotrophins).

Whereas firm conclusions are prevented by the use of male animals only, variable reporting quality and unclear risk of bias in many studies, our results suggest that aerobic exercise is a promising tool in the management of focal neuropathic pain. Registration PROSPERO CRD42021231286.

Perspective: This systematic review and meta-analysis demonstrates that aerobic exercise reduces neuropathic pain-related behavior in preclinical models of sciatic nerve injury. This effect is accompanied by changes in biomarkers associated with inflammation and neurotrophins among others. These results could help to develop exercise interventions for patients with neuropathic pain.

## 1 Introduction

Neuropathic pain is defined as pain caused by a lesion or disease of the somatosensory nervous system.^74^ It is estimated that 7% to 10% of the European population experiences neuropathic pain, equivalent to ~50 million people.^78^ Various causes of neuropathic pain have been described, including trauma, infections, metabolic abnormalities, neurotoxicity or mechanical nerve compression.^81^ Compared to pain of nociceptive origin, neuropathic pain is associated with higher pain severity, more impaired quality of life and higher health care costs.^63^ Pharmacology remains the first-line treatment for neuropathic pain. However, currently available pharmacological options provide only limited benefit often with significant side effects.^28^

In addition to pharmacology, physiotherapy and, in particular, exercise is recommended in clinical guidelines for focal^46^ and systemic^40^ neuropathic pain. However, evidence for the benefit of exercise for people with neuropathic pain is still limited. Whereas exercise seems promising in reducing symptoms in people with chemotherapy induced neuropathy, findings remain equivocal for ‘sciatica’, one of the most common entrapment neuropathies.^29^ Of note, study heterogeneity is high with a wide range of physiotherapeutic exercises studied (e.g., strength, aerobic, balance) and differing doses (e.g., duration, intensity). This variation reflects the lack of consensus on which type or dose of exercise may be most promising for people with neuropathic pain.

Exercise as a treatment for neuropathic pain and nerve injury has gained increasing interest in preclinical research. This literature has explored potential mechanistic effects of exercise by describing changes to biomarkers related to nerve injury and neuropathic pain. As such, the preclinical literature, which is more extensive than the available human literature, may help shed light on the optimal type of exercise to tackle neuropathic pain and modulate relevant biomarkers.^45^ Findings from the preclinical literature may thus help inform the design of optimal exercise programmes to trial in humans. The objectives of this preclinical systematic review are therefore: 1) to examine whether exercise is effective to improve neuropathic pain-related behaviours 2) to explore what type of exercise is most effective to improve neuropathic pain-related behaviours; 3) to identify what dose of exercise is most effective to improve neuropathic pain-related behaviours; and 4) to explore the mechanisms of effect of exercise by evaluating its consequences on biomarkers related to neuropathic pain. We will thereby focus on experimental sciatic injury, which is the most studied preclinical model of neuropathic pain.

## 2 Methods

This preclinical systematic review was conducted following the guidelines of the Systematic Review Center for Laboratory Animal Experimentation (SYRCLE),^37^ the Cochrane Handbook for Systematic Review of Intervention.^23^ The authors are reporting in accordance with the most recent guidelines “Preferred Reporting Items for Systematic Reviews” (PRISMA).^50^ The protocol has been prospectively registered in the International Prospective Register of Systematic Reviews (PROSPERO, CRD42021231286). No deviations from the protocol were made.

### 2.1 Literature search

A systematic search was developed following the step-by-step guide suggested by Leenaars et al.^37^ The following databases were searched without language restriction from inception to 2^nd^ of February 2021, with an update search on the 20th of November 2022: MEDLINE, EMBASE, Web of Science, PubMed, SCOPUS, CINAHL and Cochrane library. The search strategy for each database is described in the supplementary material (Supplementary Table 1) and is based on the combination of medical terms (MeSH) and keywords relating to the following concepts: population: sciatic nerve injury; animal models AND neuropathic pain; intervention: exercise; comparator; sham or control; outcome: behavioural tests and/or biomarkers.

### 2.2 Selection Criteria

#### 2.2.1 Types of studies

We included original animal studies reporting the effect of exercise interventions compared to a control group on peripheral neuropathic pain after experimental sciatic injury. Case studies, cross-over studies, and studies without a separate control group were excluded. Letters, reports, or abstracts from conferences were excluded.

#### 2.2.2 Animal models

In-vivo animal models of neuropathic pain induced by focal sciatic nerve injury (e.g., ligation, crushing or transection) were included. We excluded studies where nerve injury was followed by surgical repair (e.g., cut and repair model). We also excluded studies in animals with co-morbidities (e.g., pre-ischemic injury) and animals with systemic diseases (e.g., diabetic or chemotherapy induced neuropathy).

#### 2.2.3 Interventions

We included studies evaluating any exercise intervention (e.g., running, swimming), independent of frequency and dosage. Studies that evaluated the prevention rather than the treatment of already existing neuropathic pain were excluded. It is common that prior to the intervention animals are familiarised with the exercise regimen (e.g., swimming or treadmill). If this familiarisation was performed over a period longer than two weeks before nerve injury, the study was excluded because of potential preventative effects which albeit interesting are beyond our review questions.^16^ Studies evaluating exercise in combination with other treatments (e.g., treadmill plus therapeutic ultrasound, pharmacological or invasive treatments such as radiofrequency or spinal stimulation) were also excluded.

#### 2.2.4 Comparators

The control population was defined as a cohort of animals in which the same sciatic nerve injury was induced, but which either did not receive treatment or received a sham intervention. Studies comparing exercise interventions to substantive control interventions such as pharmacology or passive treatment (e.g.: passive joint mobilizations) were excluded.

#### 2.2.5 Outcome measures

Studies were included if they reported on the effect of the exercise interventions on behavioural tests (primary outcome measure) and/or biomarkers related to neuropathic pain or nerve injury (secondary outcome measure). Neuropathic pain-related tests could include stimulus-evoked methods (e.g., von Frey filaments, Hargreaves, Randall Selitto) or non-stimulus evoked methods (e.g., conditioned place preference test, grimace scale, burrowing). Biomarkers of neuropathic pain could include a wide range such as markers related to the immune and opioid systems, neurotrophins and neurotransmitters or ion channels.

### 2.3 Study selection

After the database search, duplicates were identified with MENDELEY© and removed. Unique articles were imported into the Rayyan application^49^ to facilitate screening. In the first stage, two reviewers (L.M. and C.B.) independently assessed the eligibility of the identified studies based on information from title, abstract and keywords. During the second stage, the remaining full text articles were again independently reviewed for eligibility by both reviewers (L.M. and C.B.). A third reviewer (A.S.) acted as a mediator if consensus was not reached at both title/abstract and full text screening stages.

### 2.4 Data extraction and management

Data of eligible studies were extracted by three reviewers (L.M, C.B. and J.F.) into an excel file, assuring that data for each study was independently extracted by two different reviewers. Extracted data included bibliographic information (first author, year of publication), animal characteristics (species, age, weight, and sex) and sciatic nerve injury model. We also extracted information on treatment and control groups and intervention characteristics (type of exercise intervention, timing of intervention, number of treatment sessions, duration and intensity). The duration of exercise was calculated as the total minutes exercised during the treatment period (duration_total_) as well as the total minutes exercised divided by the treatment duration in days (duration_per day_). Due to the heterogeneity of exercise regimens, grading of intensity was challenging and we thus adopted a pragmatic approach to classify exercise intensity as low, medium or high. First, we used the physiological variable of Vo2 max as a variable of intensity. ^53^ In the absence of rodent data, we used human-derived cut-offs of >88% of Vo2 max as high, between 66% and 88% as medium and under 66 %Vo2 max as low. ^58^ Second, we used the author-declared intensity if available in text as high, medium or low. When not available, we classified intensity based on the treadmill speed or percentage of inclination. Based on data from running stress tests in rats,^15^ we defined a speed of over 27m/min and/or more than 8% inclination as high intensity, between 11m/min and 26m/min and/or 1-8% inclination as medium intensity, and less than 11m/min and/or no inclination as low intensity. If inclination and speed were not in the same intensity classification, the higher intensity classification was used in the analysis. To classify the intensity of swimming interventions, we used information about external loads added to animals. Voltarelli et al.^80^ estimated the anaerobic threshold to be at 5% overload of the animals’ weight. We considered overloads ≥5% as high intensity, 0.5 to 4.9% as medium intensity and no additional load as low intensity.

For outcome measures, we extracted the type and time-point of pain-related behavioural tests and biomarkers including in which tissue and with which method they were measured. We therefore chose the closest time-point to the end of treatment to reflect effects associated with the longest possible intervention. We attempted to extract means, standard deviations, sample sizes and p-values for behavioural tests and biomarkers. If data were only available in graphs, we extracted data using the Web Plot Digitizer online version (apps.automeris.io/wpd/).^54^ Accuracy of the double extracted data was checked, and consensus reached between investigators. In case of disagreement, a third investigator (A.S.) made the final decision.

For biomarker analysis of neuropathic pain, we grouped them into the following broad categories: a) Immune system (e.g., CD68, CD3, GFAP, cytokines); b) Neurotrophins (e.g., NGF), c) Opioid pathways (e.g., β-endorphins, MOR); d) Neurotransmitters (e.g., substance P); e) ion channel and vesicles (e.g., TRPV1, TRPV8); f) transcription factors (e.g., FosB); and g) others.

### 2.5 Methodological quality assessment

#### 2.5.1 Risk of bias assessment

The risk of bias of each included study was assessed using the SYRCLE’s risk of bias tool^25^ scored by two independent reviewers (L.M. and C.B.) The reviewer with experience in using the SYRCLE tool (L.M.) trained the second reviewer in its application. Both reviewers scored 10 papers independently and discussed results to standardise application of the tool. Consensus was reached on the tenth item “others” to include items such as replacement of dropouts. Disagreement or discrepancies were resolved by a third reviewer (A.S.).

#### 2.5.2 Reporting quality

The quality of each study was assessed using the CAMARADES tool ^59^ scored by two independent reviewers (L.M and C.B.). Training in the application of the CAMARADES tool was comparable to the SYRCLE tool described above. We took a pragmatic approach to interpret the scale, since not all items were relevant to this review. Item 7 (animals with hypertension or diabetes) was not rated, so that a maximum of 9 points could be reached. Any disagreement or discrepancy were resolved by a third reviewer (A.S.).

### 2.6 Data analysis and synthesis

As pre-specified in the protocol, we performed three main analyses. To answer the first question (is exercise effective to improve neuropathic pain behaviour), we performed overall meta-analyses of included studies. separate meta-analyses were performed for each behavioural measure using Review Manager 5.4^73^ where behavioural data were available for the same outcome measure at the end of the intervention from at least two studies using similar assessment methodology. For continuous data, group means at the end of the intervention, standard deviations and sample sizes were used to calculate standardised mean differences (SMD) with 95% confidence intervals (CI). Whenever a control group served more than one experimental group, we divided the total number of animals in the control group by the number of treatment groups served.^24^ We used random-effects models and inverse variance weighting methods for pooled-effect estimates, which considers the variation between studies and weighs each study accordingly. Statistical significance was set at P<0.05. Between-study heterogeneity was determined with I^2^ statistics. I^2^ values were interpreted as 0-40% as might not be important, 30-60% as moderate heterogeneity, 50-90% as substantial heterogeneity and 75-100% as considerable heterogeneity.^24^

To answer the second question (which type of exercise is most effective to improve neuropathic pain behaviour), we performed a subgroup meta-analysis evaluating and comparing the effect of different types of exercises. Studies were grouped into those using treadmill running, swimming and other types of exercise. Meta-analyses were performed as described above, and we used Holm-Bonferroni correction to adjust for multiple analyses.

To answer the third question (most effective dose of exercise), we performed univariate meta-regressions in R^22^ for the studies examining aerobic exercise (swimming, treadmill running) assessing the effect of exercise duration (duration_total_ and duration_per day_) and the exercise intensity (low, medium, high) on behavioural outcomes. Dose and intensity of other exercises could not be estimated and were therefore not included in this analysis.

Due to the high heterogeneity of reported biomarkers, meta-analysis could not be carried out for our 4^th^ question (effects of exercise on biomarkers of neuropathic pain) due to variation in anatomical measurement sites, measurement methods (e.g., gene expression, protein level, immuno-staining), and missing summary statistics in many studies. Instead, we used heat maps to report these findings for each biomarker separated by anatomical location (e.g., peripheral nerve, dorsal root ganglia, spinal cord, brain, blood). Colour coding was assigned according to the number of studies reporting changes in individual biomarker expression (e.g., increase, decrease or no change) after intervention.

Results that could not be meta-analysed or added to the heat maps are reported narratively.

To assess publication bias, funnel plots (effect size versus sample size-based precision estimate) were generated as recommended by Zwetsloot et al.^86^ Small study bias was assessed by visual inspection of funnel plot symmetries. Trim and fill analysis was performed in IBM SPSS Statistics (Version 28) to impute theoretically missing studies on the left-hand side of the plot to recalculate the overall effect size. We also performed Egger’s regression to examine publication bias.

## Results

### 3.1 Study Selection

**Figure 1:**
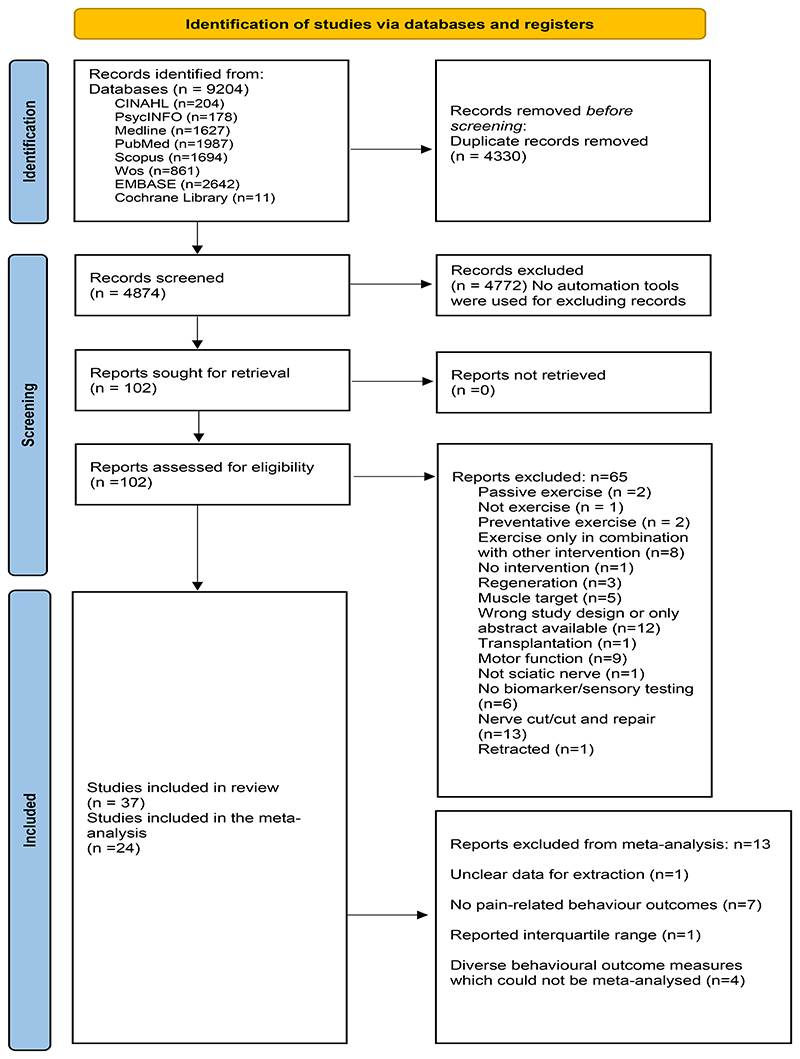
Flow diagram of study selection and inclusion

The database search retrieved a total of 9,204 articles. Following removal of duplicates, 4,874 articles were screened for titles and abstracts,102 full text studies were assessed for eligibility. Of those, 65 were excluded because they did not satisfy the eligibility criteria. This resulted in the inclusion of 37 full-text articles. The flow diagram is shown in Figure 1.

### 3.2 Risk of Bias Analysis

The majority of studies had a low risk of bias for allocation sequence generation, comparability of groups at baseline and selective outcome reporting. However, detection bias, attrition bias, and performance bias remained unclear in the majority of studies (Table 1).

#### Reporting Quality according to CAMARADES

All studies were published in peer-reviewed journals, included a statement of compliance with regulatory requirements and made an appropriate selection of anaesthetics. In contrast, only one article reported a sample size calculation, allocation concealment was mentioned in six studies (16%) and assessment blinding in 13 studies (35.4%, Supplementary Table 2).

### 3.4 Study Characteristics

Characteristics of the included articles such as details of animal species, neuropathic pain models and exercise groups are detailed in Table 2.

The most widely used model of sciatic nerve injury was nerve crush (15 studies, 40%), followed by chronic constriction injury (13 studies, 35%). Other models used were partial nerve ligation (eight studies, 21%), nerve transection (two studies, 5%) and compression injury (one study, 3%). Rats were the most prevalent species studied (25 studies, 65%) followed by mice (11 studies, 30%). Only one study with rabbits was included and another study used both rats and mice. All studies included only male animals.

The majority of studies included aerobic exercise (35 studies, 94%), 19 studies (53%) reported treadmill running (84% forced, 16% voluntary) and eight studies (23%) used swimming as the main intervention (100% forced). Three studies used a voluntarily free wheel. One study made the animals walk through a tube to eat and drink. One article directly compared swimming versus running, in both cases the exercise was forced. Three studies (8%) used other interventions (jump, balance, stairs).

To examine neuropathic pain behaviour, the majority of studies used stimulus-evoked assessments. The most common test used was mechanical hypersensitivity (von Frey hairs, 22 studies, 59%) followed by heat hypersensitivity (15 studies, 40%), cold hypersensitivity (four studies, 11%) and Randall-Selitto test (1 study, 2.7%). One study evaluated antalgic gait on a moving metallic cylinder. Only one study used spontaneous behaviours in the form of escape behaviour.

The main biomarkers reported in the studies were related to the immune system (42%) followed by neurotrophic factors (19%), neurotransmitters (17%), opioid system (3%), and ion channels (3%).

### 3.5 Effect of exercise on neuropathic pain behaviour

#### 3.5.1 Overall meta-analyses

**Figure 2:**
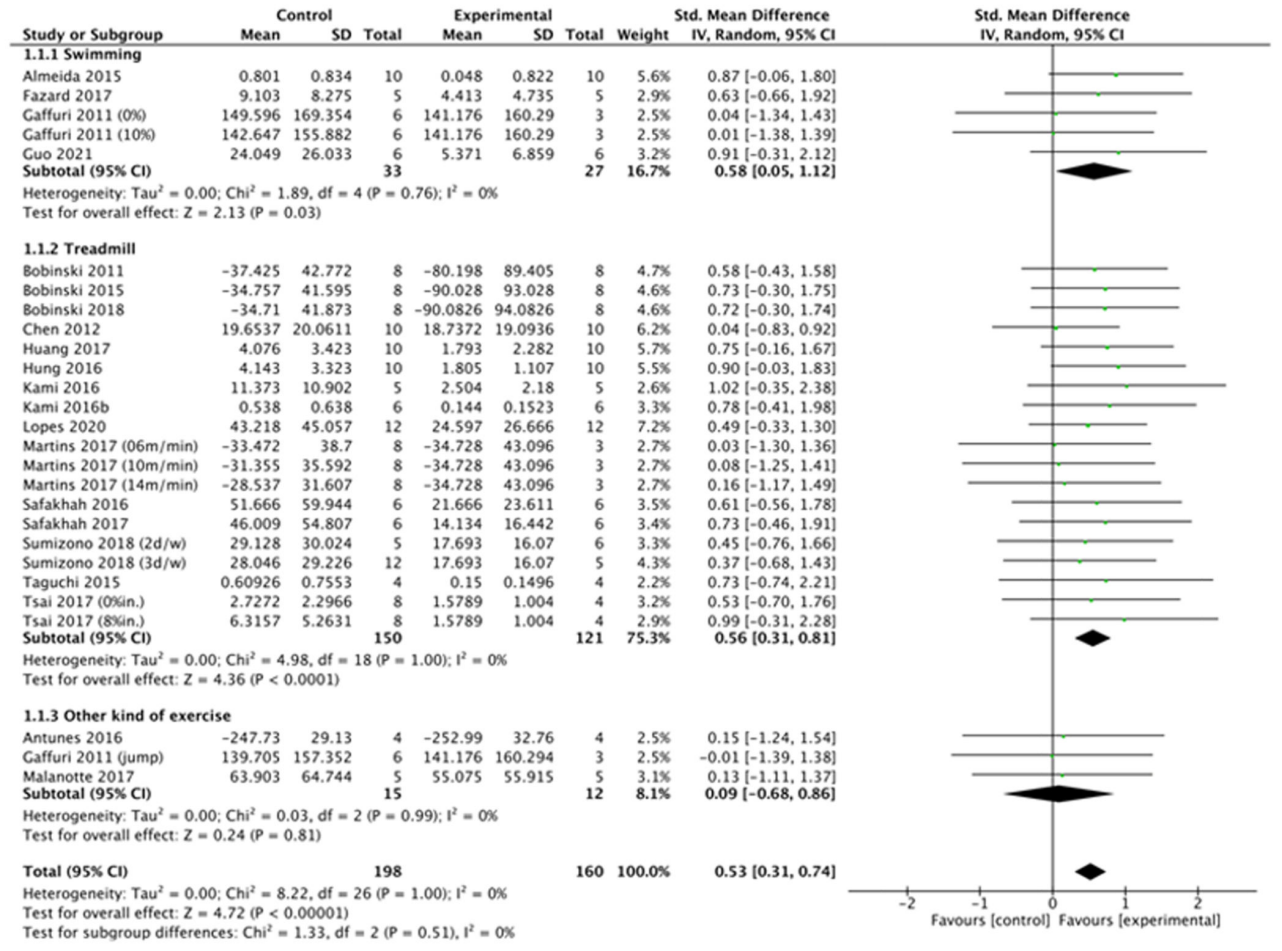
Meta-analysis of the effect of exercise on mechanical hypersensitivity

**Figure 3:**
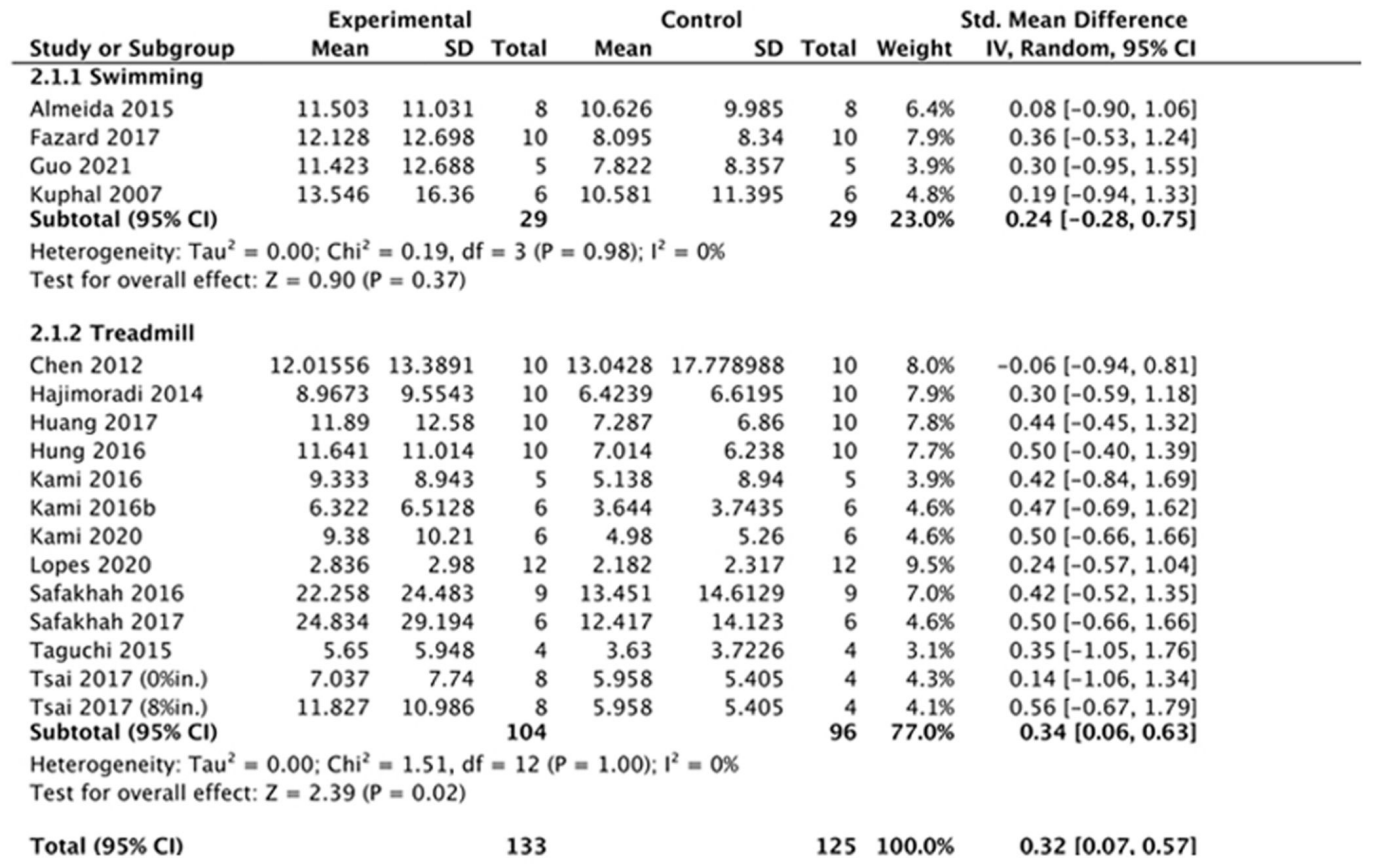
Meta-analysis of the effect of exercise on heat hypersensitivity

**Figure 4:**
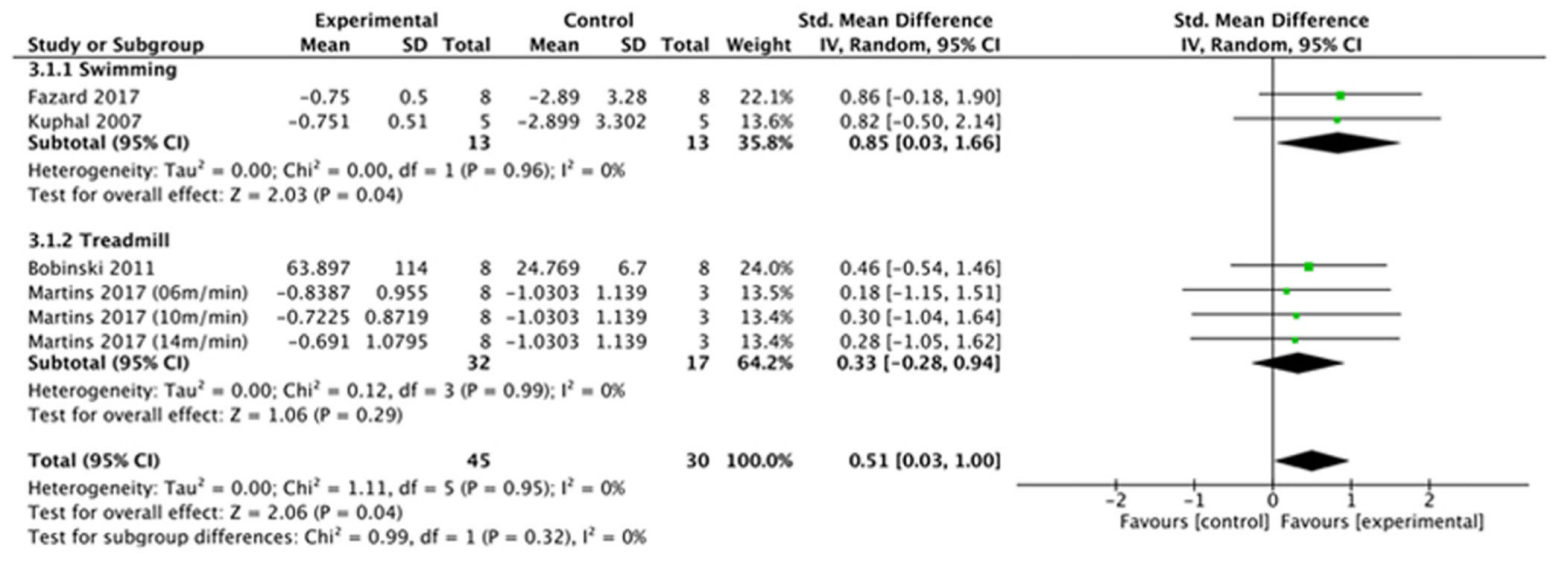
Meta-analysis of the effect of exercise on cold hypersensitivity

21 studies (n=358 animals; n=160 exercise and n=198 controls) reported on the effect of exercise on mechanical allodynia and could be included in the overall meta-analysis (Figure 2). Exercise reduced mechanical allodynia compared to control interventions (SMD (95% CI) 0.53 (0.31 to 0.74), p<0.00001, I^2^=0%). Sixteen studies (268 animals; 133 exercise and 125 control) and four studies (75 animals; 45 exercise and 30 control) could be included in the overall meta-analysis on the outcome of heat hypersensitivity (Figure 3) and cold hypersensitivity respectively (Figure 4). Exercise was superior to control interventions in reducing both heat hypersensitivity (SMD (95% CI) 0.32 (0.07 to 0.57), p= 0.01, I^2^=0%) and cold hypersensitivity (SMD (95% CI) 0.51 (0.03 to 1.0), p=0.04, I^2^=0%). Heterogeneity was ‘not important’ for the overall meta-analyses.

Six studies could not be meta-analysed; however, the majority (5, 83%) confirmed the results of our main analysis. In these studies, aerobic exercise seemed to reduce mechanical^4,35,61,82,84^ and heat hypersensitivity^61^ as well as improve escape behaviour.^8^ Only one article did not report changes after exercise treatment on mechanical hypersensitivity (Table 2).^79^

#### 3.5.2 Subgroup analysis for type of exercise

The subgroup analysis based on type of exercise demonstrated that treadmill exercise was effective compared to control interventions in reducing mechanical hypersensitivity (SMD (95% CI) 0.56 (0.31 to 0.81), p<0.0001, I^2^=0%, Figure 2) and heat hypersensitivity (SMD (95% CI) 0.34 (0.06 to 0.63), p=0.02, I^2^=0%, Figure 3), both of which survived Holm-Bonferroni correction. Swimming was effective in reducing mechanical hypersensitivity (SMD (95% CI) 0.58 (0.05 to 1.12), p= 0.03, I^2^=0%, Figure 3), although this did not survive Holm-Bonferroni correction. Swimming was not better than control interventions in reducing heat hypersensitivity (p=0.37). Other types of exercises were not superior to control interventions in reducing mechanical and heat hypersensitivity (Figure 3).

For cold hypersensitivity, the subgroup analysis for the different types of exercise (swimming, treadmill) showed superior effects compared to control for swimming (SMD (95% CI) 0.85 (0.03, 1.66), p=0.04, I^2^=0%) but this did not survive Holm-Bonferroni correction. Treadmill was not superior compared to control (p=0.29, Figure 4).

Tests for subgroup differences were not significant for mechanical and thermal hypersensitivity (p>0.32).

#### 3.5.3 Meta-regression exploring the influence exercise dose

The univariate meta-regression for the influence of exercise dose on outcome did not show any association for different doses of exercise (duration_total_ (18 studies), duration_per day_ (18 studies), intensity (18 studies)) on behavioural outcomes (p> 0.05).

Only a few studies compared different parameters of exercise in the same study. Sumizono et al.,^67^ directly compared two versus three days of exercise per week. Both frequencies reduced mechanical hyperalgesia with no differences between frequencies. Wakaizumi et al., ^82^ compared two different speeds: low (6 m/min) vs high (12 m/min). Although they found exercise reduced mechanical hypersensitivity with both speeds, no differences were reported between groups. In contrast, Martins et al., ^43^ included treadmill running at three different speeds: 6 m/min, 10 m/min and 14 m/min on a -16° slope. Whereas a trend towards a dose dependent antinociceptive effect was apparent in the early stage of the exercise intervention, no such differences were apparent at the end of treatment time-point. In addition, Tsai et al., ^75^ compared treadmill running at two types of inclination: 0% and 8%. They found that exercise at both inclinations was effective in reducing mechanical and thermal hypersensitivity. Running at 8% inclination induced a more pronounced hyposensitivity than at 0% inclination. In summary, the studies that could not be meta-analysed^43,75^ seem to confirm the results of our meta-regression that the antinociceptive effects are not necessarily associated with exercise dose. A summary of the types of exercise, duration and intensity are reported in Supplementary Table 3

### 3.6 Effect of exercise on biomarkers of neuropathic pain

Exercise seems to be effective in modulating biomarker concentrations, predominantly related to the immune system (Table 3 and Supplementary Table 4). The studies reporting on biomarkers of the immune system found a reduction of pro-nociceptive markers (e.g., TNF-α, IL-1β) at the site of the nerve lesion and also in the spinal cord. In addition, an increase of anti-inflammatory markers (e.g., IL-10) was measured after exercise. The analysis of neurotrophic factor showed contradictory results; some studies reported changes (increase or decrease) at different anatomical levels while others reported no changes. Neurotransmitters, the opioid system and ion channels had varying findings from only a few reports.

### 3.7 Publication bias

Visual inspection of funnel plots showed no asymmetry (Supplemental Figure 1), however small number of studies in the thermal hypersensitivity analyses prevent firm conclusions. Egger’s regression did not indicate effects of small studies in all three analyses (P>0.475). Trim and fill analysis did not impute any theoretically missing studies for mechanical and cold hypersensitivity. For heat hypersensitivity, three theoretically missing studies were imputed, reducing the effect size only marginally from 0.32 (95% CI 0.07 to 0.57) to 0.29 (95% CI 0.06 to 0.52).

## Discussion

Our review included 37 studies with 717 animals. Reporting quality was good but risk of bias was unclear in most included studies. Most studies included aerobic exercise (swimming and treadmill) with few studies exploring strength or coordination training. The results from the overall meta-analysis suggest that exercise is an effective treatment for reducing mechanical, heat and cold hypersensitivity. Treadmill was particularly beneficial in reducing mechanical and heat hypersensitivity. In contrast, no benefit of treadmill exercise was observed on cold hypersensitivity. These results are further supported by the studies that could not be meta-analysed. The antinociceptive effect seemed unrelated to exercise duration or intensity. Exercise seems to exert its beneficial effects by modulating a wide range of biomarkers. The main type of biomarkers reported and modulated by exercise relate to the immune system followed by neurotrophins.

### Exercise has an antinociceptive effect

Our findings on the beneficial antinociceptive effects of exercise are in broad agreement with previous preclinical work. A recent systematic review that included a range of peripheral nerve injury models (e.g., diabetic neuropathy, sciatic nerve injury) limited to rats also found mechanical and thermal antinociceptive effects of exercise.^19^ Similarly, a systematic review by Palandi et al.^51^ reported that aerobic exercise improved mechanical and thermal hypersensitivity in models of neuropathic pain after spinal cord injury.^48,60,5,47^

Understanding the hypoalgesic effects of aerobic exercise in humans has become more popular in the last decade. Various systematic reviews confirm that aerobic exercise induces heat and mechanical hypoalgesia in healthy volunteers.^47,83^ Of interest, the effect of aerobic exercise is less pronounced for cold than mechanical hypoalgesia in healthy people. Similarly, a systematic review found that aerobic exercise reduced pain sensitization in patients with chronic musculoskeletal pain.^72^ To date, there is a dearth of literature on hypoalgesic effects of aerobic exercise in patients with focal nerve injury and neuropathic pain.

### The optimal type of exercise remains to be determined

Our sub-group analysis indicated that aerobic exercise seems most suited to reduce neuropathic pain behaviours in pre-clinical models of sciatic nerve injury. It should be noted though that subgroup differences were not significant and whereas 16 studies could be analysed for treadmill running^6–8,11,26,27,30–32,39,43,56,57,67,69,75^ only five articles could be meta-analysed for for swimming^1,13,14,18,36^ and three for other types of exercise.^2,14,41^ These small numbers for swimming and ‘other exercises’ prevent firm conclusions. Guo et al.^19^ also did not find one type of exercise superior to the other, in a systematic review limited to rat models.

In humans, most investigations comparing the hypoalgesia effects of different types of exercises are performed in healthy participants following a single bout of exercise and reveal contradictory results. Wewege et al.^83^ reported a strong correlation between hypoalgesia and aerobic exercise while dynamic resistance training and isometric exercises showed a small or no correlation, respectively. In contrast, the earlier work from Naugle et al.^47^ suggests that aerobic, dynamic resistance and isometric exercise all induced hypoalgesia, with the latter producing the largest effects. In the management of patients with sciatica, a recent expert recommendation concluded that motor control, aquatic therapy, stabilising movements and isometric exercise for the trunk and the lower body are the recommended treatment options.^85^ Aerobic exercise was not included, likely due to the absence of any randomised clinical trial evaluating the effect of aerobic exercise for people with sciatica.

The slightly more extensive literature for patients with systemic neuropathies suggests that balance, endurance training and specific nerve gliding exercises could be beneficial in the treatment and prevention of neuropathy.^20,66^ However, as most studies focused on function rather than neuropathic pain, it remains unclear whether these results can be extrapolated to patients with painful focal nerve injuries. More preclinical and clinical research is greatly needed to identify the most promising types of exercises to reduce neuropathic pain after focal nerve injury.

### The analgesic effect seems independent of exercise dose

Our analysis was not able to establish a relationship between the antinociceptive effects and the duration or intensity of exercise. These results are in line with a systematic review limited to rat nerve injury models.^19^ This is intriguing since the human literature seems to indicate a correlation of the intensity^48,77^ and duration^52^ of aerobic exercise with its immediate hypoalgesic effects. However, these studies were performed predominantly in healthy participants with only few studies in patients with chronic musculoskeletal pain and none in neuropathic pain. In the absence of clear guidelines and reporting, we employed a pragmatic approach to extrapolate the intensity from the physiological variables used in the preclinical studies. Only four articles specified the intensity of the exercise performed,^6–8,60^ two described the intensity through the metabolic index^9,35^ and one used % of Vo2 max.^39^ This heterogeneity of methodology and reporting prevents the identification of exact intensities and may have contributed to our null results. In addition, factors such as species, sex and type of exercise (e.g., stress induction with forced exercise) may influence exercise affinity and exercise dose. In our data, species did not seem to majorly influence antinociceptive effects (Supplemental Figures 2-4).

Of all included studies that compared different exercise doses,^14,38,67,75,82^ only one study found a significant difference in analgesic effects.^38^ Unfortunately, the poor reporting of exercise dose and the resulting inability to identify the best dose also remains a major challenge in humans.^21^

### Exercise modulates biomarkers of neuropathic pain

Despite methodological limitations preventing biomarker meta-analysis, our review identified clear modulation of biomarkers implicated with neuropathic pain through exercise, particularly regarding the immune system.

Exercise seems to modulate neuroinflammatory markers at multiple levels of the neuraxis. This is important, since neuroinflammation has been implicated with neuropathic pain in patients.^64^

The second most studied biomarkers are the neurotrophins (e.g, NGF, BDNF), which are also strongly implicated with neuropathic pain.^62^ In humans, exercise has been shown to elevate systemic BDNF at least temporarily,^34,68^ however an association with neuropathic pain has not been explored. Our preclinical results reveal a potentially similar beneficial effect in neuropathic pain.

### Limitations

The findings from this systematic review should be interpreted with some caution. Only a small number of reports were available for meta-analysis particularly of swimming and ‘other types of exercise’ subgroups, thus limiting interpretability.^10^ Most importantly, the high percentage of studies with unclear risk of bias and poor reporting prevents firm conclusions. Unfortunately, this is a well-known challenge with preclinical research and reporting guidelines were thus published in 2010^33^ and updated in 2020.^12^ Only five included studies were published before these guidelines and hence there is no excuse for the low-quality reporting. Another consideration is the methodological (e.g., length and intensity of intervention, study designs) and biological heterogeneity (e.g., animal species, strain, neural injury type). Of note, all articles included in this review used male animals only even though neuropathic pain behaviour and the underlying mechanisms vary according to sex.^3,42,55^ Voluntary running behaviour also differs, with male rats being less active on treadmills than female rats.^5^ Importantly, women represent a large proportion of people with neuropathic pain from focal nerve injury.^44,70,71,76^ This hinders the generalisability of our findings and further highlights the urgent need of incorporating female and male animals in preclinical pain research.

We performed our meta-analysis at the closest time-point to the end of treatment. Whereas this is clinically most relevant reflecting effects associated with the longest possible intervention, we may have missed transient antinociceptive effects at earlier time points. Most data included in the meta-analyses had to be extracted from graphs, which may have introduced small measurement errors. However, the overall low heterogeneity among studies substantially increases the confidence in our results. Lastly, publication bias remains an issue particularly in preclinical studies.^17^ Reassuringly, our analyses suggested that publication bias has not majorly influenced our results. However, the analyses related to thermal hypersensitivity were based on a small number of studies, limiting interpretability.

### Clinical implications

Whereas the efficacy of aerobic exercise has been studied and found to be promising for patients with systemic neuropathies,^20,65^ such data are not available for patients with focal neuropathies such as sciatica.^85^ This may be a missed opportunity, as the preclinical data clearly suggest that exercise and in particular treadmill running is a promising therapeutic tool in the treatment of neuropathic pain after peripheral sciatic nerve injury. Presumably, high pain intensities and functional limitations particularly in the acute stages of focal nerve injuries may have prevented the inclusion of aerobic exercise in management strategies. Given the absence of a clear relationship between the dose of aerobic exercise and analgesia, clinical exercise regimens may be adjusted to an individual’s ability (e.g., arm ergometer for patients with acute lower limb pain), assuring adequate tolerability and adherence. Careful research is required to evaluate the efficacy and safety of such interventions.

## Conclusion

This systematic review and meta-analysis indicate that exercise improves neuropathic pain behaviours in preclinical models of focal nerve injury. Aerobic exercise, specifically treadmill training, demonstrated improvements in mechanical and thermal hypersensitivity. No relationship was apparent between exercise duration or intensity and antinociceptive effects. Exercise seems to exert its beneficial effect through modulation of neuropathic pain biomarkers at different sites of the neuraxis. Whereas firm conclusions are prevented by the use of male animals only, poor reporting quality and unclear risk of bias in many studies, these results encourage translation to patients and future research into the safety and benefit of exercise in patients with focal nerve injuries.

## Supplementary Material

Supplementary Table 1

Supplementary Table 2

Supplementary Table 3

Supplementary Table 4

Supplemental Figures

## Figures and Tables

**Table 1:** Risk of Bias

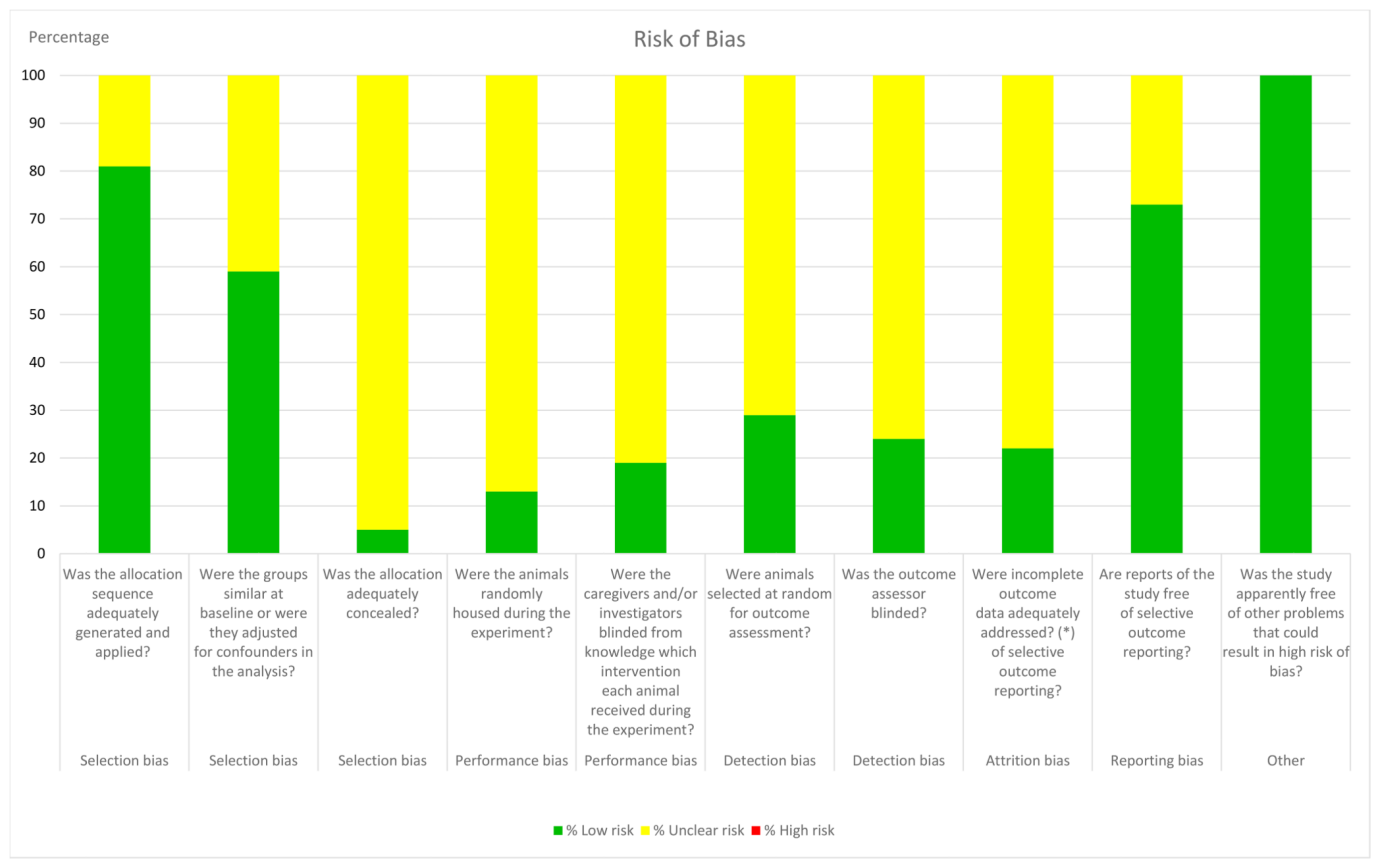

**Table 2:** Characteristics of included studies

Reference	Animals	Model	Groups (bold denotes meta-analysed groups)	Intervention (type, start, intensity, frequency)	Behavioural tests
Almeida 2015	BALB/c mice, male, 23.5-60.24g	Partial nerve ligation (PNL)	**PNL (n=10) PNL+Swimming (n=10)** PNL+Swimming+Detraining (n=10) sham-operated and sedentary animals(n=10)	**Swimming:** POD7: 3 sessions of 10 min, 2 of 20, 30, 40 minutes and 13 of 50 minutes. 25 session, 5 sessions/week 2 day rest	Von Frey Plantar test
Ashour 2017	albino rats, male, adult, 120- 150g	Nerve crush injury (NCI)	Sham surgery (n=8) **NCI (n=8)** **NCI+Swimming (n=8)**	**Swimming:** POD ?: Increasing duration (20-90 min) from day 1 to 5 with 1-2 rest periods (10min) in total	not included
Antunes 2016	Wistar rats, male,10 weeks, 352g	Siciatic nerve compression (NC	Not operated (n=4) **NC (n=4)** **NC + strength (n=4)**	**Strength**: POD 3: wooden vertical ladder with 67 iron steps (height: 1.18 m, width: 20.5 cm, inclination: 60°). Dark box positioned at top to rest after one bout of exercise, series of 10 ladder ascents with 100 gram load on tail,1 min rest.	Von Frey
Bertolini 2011	Wistar rats, 412.6 +- 49.27g	Chronic constriction injury (CCI	**CCI (n=6)** **CCI+Low intensity (n=6)** **CCI+Progressive-time (n=6)**	**Swimming:** POD ?: 10 min per session progressing to 60 min in last week (6 weeks)	Moving metallic cylinder
Bobinski 2011	Swiss mice, male, 8-9 weeks old,25-35g	Nerve crush injury (NCI)	**NCI (n=8)** **NCI+Treadmill (n=8)**	**Low-intensity aerobic treadmill:** POD 3: 30 min at 10m/min without inclination, 5 days per week for 2 weeks	Von Frey Cold plate test
Bobinski 2015	Swiss mice, male, 8-9 weeks old,25-35g	Nerve crush injury (NCI)	**NCI (n=8)** **NCI+Exercise (n=8)**	**Low-intensity aerobic treadmill**: POD 3: 30 min at 10m/min without inclination, 5 days per week for 2 weeks	Von Frey
Bobinski 2018	Swiss mice, male, 20-30g	Nerve crush injury (NCI)	**NCI (n=8)** **NCI+Treadmill (n=8)**	**Low-intensity aerobic treadmill:** POD 3: 30 min at 10m/min without inclination, 5 days per week for 2 weeks	Von Frey
Bonetti 2015	Wistar rats, male, 3-month, 280–330 g.	Nerve crush injury (NCI)	Sham surgery (n=5) **NCI (n=6)** **NCI+Endurance (n=6)** **NCI+Balance and coordination (n=6)**	**Endurance**: POD 2: treadmill: 20 min on the first day, progressively increasing every day up to 50 min on the fifth day and then 60 min for the next 4 weeks. Warm-up: 5 min at 30% of the maximum speed reached in the MET (5.5 m/min), 10–50 min at 45–55% (9 m/min), and 5 min of recovery at 30% (5.5 m/min). 5 sessions/week for 5 weeks **Balance/coordination:** 5 different elevated obstacles per day with increasing difficulty. Obstacles included suspension bridges, rope bridges, and parallel bars.	not included
Byun 2005	Sprague-Dawley rats, male, 6 weeks, 200+/-10g	Nerve crush injury (NCI)	Sham surgery (n=8) **NCI (n=8)** **NCI+Running (n=8)**	**Treadmill:** POD 3: 30 min once a day for 12 days, 8m/min, 0% inclination	Not included
Chen 2012	Sprague-Dawley rats, male, 250-300g	Chronic constriction injury (CCI)	**CCI(n=10)** **CCI+Swimming (n=10)** **CCI+Treadmill (n=10)**	**Swimming**: POD 1: starting with 9 sessions of 10 min, 15 min rest increasing to day 7: 1 session of 90 min without rest. 39 weeks, 5 days per week **Treadmill:** POD 1: starting at 1.2 km/h for 15-30 min increasing to 1.8 km/h for 30 min at 2 weeks. From week 3 to 6 running at 1.8 km/h for 60 min, 5 days per week	Von Frey Hargreaves
Fazard 2017	Wistar rats, male, adult, 180–220 g.	Chronic Constriction Injury (CCI)	Naïve (n=10) Sham surgery (n=10) **CCI(n=10) CCI+Swimming (n=10)**	**Swimming:** POD 3: 10-minute bouts of swimming interspersed with five-minute rest periods. 5 times/week over 4 weeks	Randall-Selitto Von Frey Cold Allodynia Hargreaves
Gaffuri 2011	Wistar rats, male14 weeks, 393.70 ± 30.37g	Chronic Constriction Injury (CCI)	**CCI+Placebo Group(n=6) CCI+Swimming no load (n=6) CCI+Swimming 10% load (n=6) CCI+Jump Group(n=6)**	**Placebo: composed of animals submitted to swimming less than one minute** **Swimming:** POD ?: 30 minutes swimming using no or 10% body overload. Five sessions with rest intervals of 48 hours **Jump:** POD ?: overload of 50% of the animal’s weight. Four sets of 5 jumps each with 30 sec between sets. Five sessions, with rest intervals of 48 hours	Von Frey
Guo, 2021	Sprangue-Dawley rats, male, adult, 200-220 g	Chronic constriction injury (CCI)	**CCI (n=6) CCI+Swimming (n=6)**	**Swimming:** POD 3: 2 sessions of 30 min, 2 sessions of 45 min and 13 sessions of 60 min. Every 3 days. the animals had a rest day.	Von Frey
Hajimoradi 2014	Wistar rats, male, adult, 250- 300 g	Nerve crush injury (NCI)	**NCI (n=10) NCI+Treadmill (n=10)**	**Treadmill:** POD 2: once a day for 30 min (constant inclination of 0, 17 m/min) for 21 days.	Hot Plate
Huang 2017	Sprague-Dawley rats, male, 220-270g	Chronic constriction injury (CCI)	**CCI(n=10) CCI+Treadmill (n=10)**	**Treadmill**: POD 8: 30 min/day for 3 weeks, 14-16 m/min 8% inclination.	Von Frey Hargreaves
Hung 2016	Sprague-Dawley rats, male, 220-270g	Chronic constriction injury (CCI)	**CCI(n=10) CCI+Treadmill (n=10)**	**Treadmill** POD 3: 14-16 m/min with 8% inclination for 30 min. 5 days a week for 4 weeks.	Von Frey Hargreaves
Kami, 2016	C57BL/6 J mice, male, 10 weeks,	Partial sciatic nerve ligation (PNL)	**PNL (n=5) PNL+Running (n=5)**	**Voluntary run wheel**: free running until day 15	Von Frey Plantar test
Kami 2016 b	C57BL/6 J mice, Adult	Partial sciatic nerve ligation (PNL)	**PNL (n=6) PNL+Running (n=6)**	**Treadmill:** POD 2: 1st week 7 m/min for 10 min/day. 2^nd^ week 7 m/min for 20-60 min/day (increase 10/day). 3^rd^ week 7 m/min for 60 min/day, 5 days/week.	Von Frey Hargreaves
Kami 2020	C57BL/6J mice, Adult	Partial sciatic nerve ligation (PNL)	**PNL (n=10) PNL+Running (n=10)**	**Running:** POD 0: free running until day 14.	Plantar Test
Korb 2010	Wistar rats, male, adult, 200- 250 g	Sciatic nerve transection (NT)	Naïve (n=5) Naïve+Treadmill(n=5**)** **NT (n=5) NT+Treadmill (n=5)**	**Treadmill:** POD?: 20 min on the first day increasing everyday up to 50 min on the fifth day. Then 60 min during the next 4 weeks. Protocol: 5 min warm-up period at 30% of the maximal speed reached in the MET (5.5 m/min), 10-50 min running at 45-55% (9 m/min) and 5 min recovery at 30% (5.5 m/min). 5 sessions/week for 4 weeks.	Von Frey
Kuphal 2007	Sprague-Dawley rats, male, 250–300 g CD1 mice, male, 30–35g	Partial sciatic nerve ligation (PNL)	Rats: **PNL (n=8) PNL+Exercise (n=8)** Mice: **PNL (n=10) PNL+Exercise (n=10)**	**Swimming**: POD 1. Increasing from 10 to 90 min with 15 min rest in the first 22 sessions. 2 sessions for 90 min without rest.	Cold plate Radiant heat
Liao 2017	Rats	Sciatic nerve transection; (NT)	**PNL (n=10) PNL+Swimming S10 (n=10) PNL+Swimming S20 group (n=10) PNL+Swimming S30 group (n=10)**	**Swimming:** POD 7. S10: 10 mins × 3 time/week S20: 20 mins × 3 time/week S30: 30 mins × 3 time/week.	Not included
Lopes 2020	Wistar rats, Male, 8 weeks old, 280 ± 20 g	Chronic constriction injury (CCI)	Naïve rats (n=12) Sham surgery (n=12) **CCI(n=12) CCI+Treadmill (n=12)**	**Treadmill**: POD 15: 70% of VO2max. The rats performed one 20-min running session daily for 8 days (08:00 - 09:30 a.m.) without inclination.	Von Frey Plantar test
Malanotte 2017	Wistar rats, male, 8 weeks, 314±23g	Nerve crush injury (NCI)	Naïve rats (n=5) **NCI (n=5) NCI+Exercise (n=5)**	**Jumping exercises:** POD 3: aquatic environment, weight overload was attached with a Velcro® strap to the back of the animal. 20 days, with 3 days of exercise followed by a one-day rest each week, and a two-day interval between weeks. 1^st^ week: 2 sets of 10 jumps. 30 sec intervals between sets. 2^nd^ week: 3 sets of 10 jumps. 30 sec intervals between sets. 3^rd^ week: 4 sets of 10 jumps. 30 sec intervals between sets.	Von Frey
Martins 2017	Swiss mice, 8 weeks old, Male, 25-30 g	Nerve crush injury (NCI)	**NCI NCI+Eccentric exercise (6 m/min) (n=8) NCI+eccentric exercise (10 m/min) (n=8) NCI+eccentric exercise (14 m/min) (n=8)**	**Eccentric Treadmill:** (Downhill Running) Program: 30 min at a speed of 6, 10, or 14 m/min with -16° slope, 5 days/week for 8 weeks.	Von Frey Cold Hyperalgesia
Safakhah 2016	Wistar rats, male mature 200±20gr	Partial sciatic nerve ligation (PNL)	Naïve rats (n=6) Sham surgery (n=6) **PNL (n=6)** PNL+**Treadmill (n=6)**	**Treadmill:** POD 3: 30 min, 5 days/week for 14 days at 16 m/min.	Von Frey Plantar Test
Safakhah 2017	Wistar rats, male, adult, 200±220g	Chronic constriction injury (CCI)	Naïve rats (n=6) Sham surgery (n=6) **CCI (n=6) CCI+Treadmill (n=6)**	**Treadmill:** POD 3: 16m/min near 70% VO2 max, 30 min/per day, 5 days/week for 3 weeks.	Von Frey Plantar Test
Seo 2006	Sprague-Dawley rats, male, 8 weeks, 220-240g	Nerve crush injury (NCI)	Control (n=6) **NCI (n=6) NCI+Treadmill (n=6)**	**Treadmill**: POD 3: 18m/min, 30 min, 2 times/day for 2 weeks.	Not included
Seo 2009	Sprague-Dawley rats, male, 7-8 weeks, 200-220g	Nerve crush injury (NCI)	Naïve rats (n=6) **NCI (n=6) NCI+Treadmill low intensity(n=6) NCI+Treadmill high intensity (n=6)**	**Treadmill**: POD 3: 18m/min, 30 min, 2 times/day for 2 weeks. low intensity: 8m/min high intensity: 36m/min.	Not included
Shen 2013	Sprague-Dawley rats, male, 250-300g	Chronic constriction injury (CCI)	Naïve rats (n=6) **CCI (n=6) CCI+Swimming (n=6)**	**Swimming**: POD 5: 5 min to 1 hour with 5 min rest, 1 day 4 times per week for 2 weeks.	Von Frey Hargreaves
Sumizono 2018	Sprague Dawley rat; male; 8 weeks; 274.3 ± 21.2 g	Chronic constriction injury (CCI)	Naïve (n=6) **CCI + high-frequency exercise (HFE, n=27)** **CCI+ low-frequency exercise** **(LFE, n=10)** **CCI + no exercise (NE, n=25)**	**Treadmill running:** POD 1-2: Speed of 20 m/min; HFE for 5 days; LFE for 3 days. Total treatment: 5 weeks Dose: 30 min/ speed was approximately 55% of maximal oxygen consumption.	Von Frey
Taguchi 2015	C57BL / 6J mice, male, 12 weeks	Partial Sciatic Nerve Ligation (PNL)	Naïve (n=4) **PSL (n=4) PSL+Treadmill (n=4)**	**Treadmill:** POD 2: 7m/min for 20 min to 60 min, 5 days for 1 week.	Von Frey Plantar test
Tsai 2017	Sprague-Dawley, male, 285–335 g	Chronic constriction injury (CCI)	**CCI** (n=8) **CCI+Treadmill 0%-incline **(n=8) **CCI+Treadmill 8%-incline **(n=8)	**Treadmill:** POD 6: 14-16 m/min with/without 8% incline for 30 min. Daily sessions for 3 weeks.	Von Frey Hargreaves
Van Meerten 1996	rats male, 140-160g	Nerve crush injury (NCI)	**NCI (n=12) NCI+Exercise (n=12)**	**Walking tube**: forced to walk 8 meters for food and water.	Foot reflex withdrawal test to electrical stimulus Hargreaves
Wakaizumi 2016	C57BL/6J mice and DAT-Cre mice, male, 7-13 weeks	Partial Sciatic Nerve Ligation (PNL)	**PNL (n=4) PNL+ Treadmill low speed (2 weeks) (n=4) PNL+Treadmill high speed (n=4)**	**Treadmill:** POD? 60 min/day, 5 days per week for one or two weeks. Max speed of 6 or 12 m/min.	Von Frey Plantar Test
Wang 2016	New Zealand white rabbit, male, 11±12 weeks-old, 1.78 ±0.12 kg	Nerve crush injury (NCI) -sciatic nerve	**NCI (n=6) NCI+Treadmill (n=6)**	**Treadmill:** POD 3: Days 1-3 at a rate of 10m/min for 20 minutes a day; Days 4-6 at a rate of 15m/min for 20 min/day; Days 7-28 at a rate of 20m/min for 20 min/day. 6 days/week	Not included
Whitehead 2017	Sprague Dawley rats, 300-425g	Chronic constriction injury (CCI)	Sham+unlocked wheel (n=8) **CCI+locked wheel(n=8) CCI+unlocked wheel(n=8)**	**Wheel free running**: POD 2: 1 hour for 18 days	Von Frey

EX: exercise; ?: not reported; MET: metabolic index; POD: post operative day; PNL: partial nerve ligation; CCI: chronic constriction injury; NCI: nerve crush injury; NC: nerve compression; NT: nerve transection, min: minutes, sec: seconds; HFE: high frequency exercise; LFE: low frequency exercise

**Table 3:** Changes in neuropathic pain related biomarkers following exercise. Heatmaps demonstrate the frequency of studies showing specific directions of biomarker modulation at different anatomical sites.

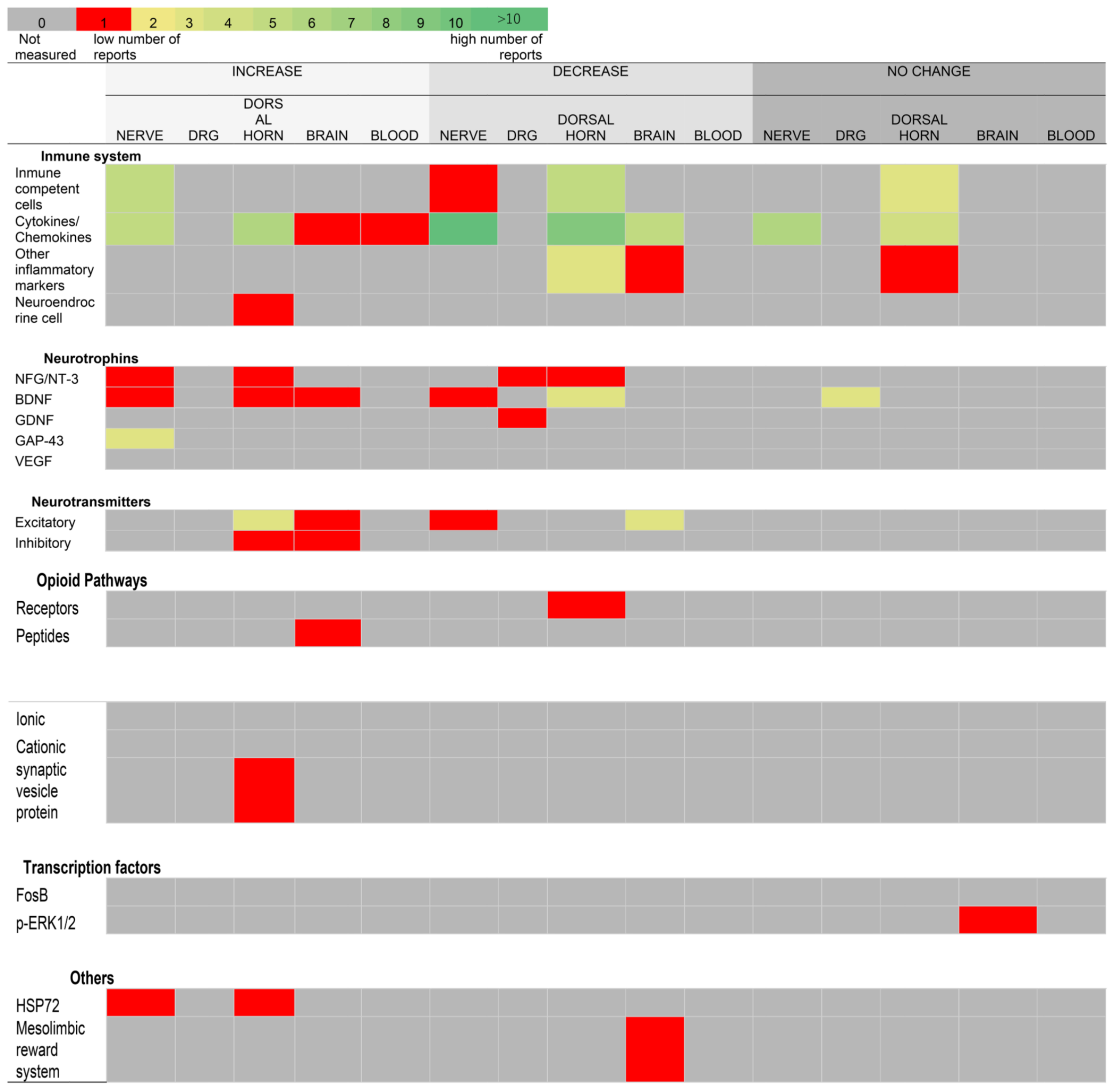

## Highlights

‘What is already known:

- Exercise is recommended in clinical guidelines for focal peripheral neuropathic pain
- Evidence for its benefit is lacking and the optimal exercise type and dose remain unknown

‘What are the new findings:

- Preclinical data suggest that exercise reduces peripheral nerve injury-induced hypersensitivity
- Duration and intensity of exercise does not seem to influence this antinociceptive effect
- Exercise modulates a range of biomarkers, particularly immune-related and neurotrophic factors
