# Supplementary material for: Effect of Type and Dose of Exercise on Neuropathic Pain after Experimental Sciatic Nerve Injury: a Preclinical Systematic Review and Meta-analysis
Source: J Pain. Author manuscript; Available in PMC 2026 Jun 17. (PMC7619194; doi:10.1016/j.jpain.2023.01.011)
Supplement: Supplementary Table 1 [file EMS213962-supplement-Supplementary_Table_1.docx]

**Supplemental table 1:** Database formulas during literature search

Total original search 2^nd^ of February 2021: 7929

Total updated search 20^th^ of November 2022: 9204

**CINAHL Search Formula EBSCO**

Original search:173

Updated search: 204

| **Search number** | **Query** | **Filters** |
| --- | --- | --- |
| **#1** | ("Models, Animal”[Mesh] OR “animal*” OR "Mice"[Mesh] OR "Rats"[Mesh] OR “transgenic mice” OR “rodent”) | No |
| AND | ("Pain"[Mesh] OR "Pain" OR "Peripheral Nervous System Diseases"[Mesh] OR "Neuropathic pain" OR “Nerve pain” OR “Neuralgia”[Mesh] OR “nerve block method*” OR “nerve crush” OR “nerve constriction” OR “nerve crush” OR “nerve cut” OR “nerve injury” OR “nerve transection” OR “nerve ligation” OR “chronic constriction injury” OR “peripheral neuropathy” OR “nerve inflammation” OR “neuropathy”) | No |
| AND | ("Sciatic Neuropathy"[Mesh] OR "Sciatica"[Mesh] OR "sciatica" OR "Sciatic Nerve"[Mesh] OR "sciatic nerve") | No |
| AND | ("Physical Conditioning, Animal"[Mesh] OR “Physical therapy modalities”[Mesh] OR “physical therap*” OR “physiotherap*” OR “Exercise”[Mesh] OR “exercise therapy” OR “exercise*” OR “sham exercise” OR “run*” OR “swim*” OR “walk*” OR “Locomotion”[Mesh] OR “strength*” OR “endurance” OR “resistance” OR “physical conditioning” OR “motor activity” OR “aerobic*” OR “isometric*” OR “isotonic*” OR “isokinetic*” OR “resistance” OR “train*” OR “treadmill” OR “vibration”) | No |

**PsycINFO Search Formula via EBSCO**

Original search: 170

Updated search: 178

| **Search number** | **Query** | **Filters** |
| --- | --- | --- |
| **#1** | ("Models, Animal”[Mesh] OR “animal*” OR "Mice"[Mesh] OR "Rats"[Mesh] OR “transgenic mice” OR “rodent”) | No |
| AND | ("Pain"[Mesh] OR "Pain" OR "Peripheral Nervous System Diseases"[Mesh] OR "Neuropathic pain" OR “Nerve pain” OR “Neuralgia”[Mesh] OR “nerve block method*” OR “nerve crush” OR “nerve constriction” OR “nerve crush” OR “nerve cut” OR “nerve injury” OR “nerve transection” OR “nerve ligation” OR “chronic constriction injury” OR “peripheral neuropathy” OR “nerve inflammation” OR “neuropathy”) | No |
| AND | ("Sciatic Neuropathy"[Mesh] OR "Sciatica"[Mesh] OR "sciatica" OR "Sciatic Nerve"[Mesh] OR "sciatic nerve") | No |
| AND | ("Physical Conditioning, Animal"[Mesh] OR “Physical therapy modalities”[Mesh] OR “physical therap*” OR “physiotherap*” OR “Exercise”[Mesh] OR “exercise therapy” OR “exercise*” OR “sham exercise” OR “run*” OR “swim*” OR “walk*” OR “Locomotion”[Mesh] OR “strength*” OR “endurance” OR “resistance” OR “physical conditioning” OR “motor activity” OR “aerobic*” OR “isometric*” OR “isotonic*” OR “isokinetic*” OR “resistance” OR “train*” OR “treadmill” OR “vibration”) | No |

**Medline Search Formula (EBSCO)**

Original search: 1149

Updated search: 1627

| **Search number** | **Query** | **Filters** |
| --- | --- | --- |
| **#1** | ("Models, Animal”[Mesh] OR “animal*” OR "Mice"[Mesh] OR "Rats"[Mesh] OR “transgenic mice” OR “rodent”) | No |
| AND | ("Pain"[Mesh] OR "Pain" OR "Peripheral Nervous System Diseases"[Mesh] OR "Neuropathic pain" OR “Nerve pain” OR “Neuralgia”[Mesh] OR “nerve block method*” OR “nerve crush” OR “nerve constriction” OR “nerve crush” OR “nerve cut” OR “nerve injury” OR “nerve transection” OR “nerve ligation” OR “chronic constriction injury” OR “peripheral neuropathy” OR “nerve inflammation” OR “neuropathy”) | No |
| AND | ("Sciatic Neuropathy"[Mesh] OR "Sciatica"[Mesh] OR "sciatica" OR "Sciatic Nerve"[Mesh] OR "sciatic nerve") | No |
| AND | ("Physical Conditioning, Animal"[Mesh] OR “Physical therapy modalities”[Mesh] OR “physical therap*” OR “physiotherap*” OR “Exercise”[Mesh] OR “exercise therapy” OR “exercise*” OR “sham exercise” OR “run*” OR “swim*” OR “walk*” OR “Locomotion”[Mesh] OR “strength*” OR “endurance” OR “resistance” OR “physical conditioning” OR “motor activity” OR “aerobic*” OR “isometric*” OR “isotonic*” OR “isokinetic*” OR “resistance” OR “train*” OR “treadmill” OR “vibration”) | No |

**PubMed Search Formula NLM**

Original search: 1822

Updated search: 1987

| **Search number** | **Query** | **Filters** |
| --- | --- | --- |
| **#1** | ("Models, Animal”[Mesh] OR “animal*” OR "Mice"[Mesh] OR "Rats"[Mesh] OR “transgenic mice” OR “rodent”) | No |
| AND | ("Pain"[Mesh] OR "Pain" OR "Peripheral Nervous System Diseases"[Mesh] OR "Neuropathic pain" OR “Nerve pain” OR “Neuralgia”[Mesh] OR “nerve block method*” OR “nerve crush” OR “nerve constriction” OR “nerve crush” OR “nerve cut” OR “nerve injury” OR “nerve transection” OR “nerve ligation” OR “chronic constriction injury” OR “peripheral neuropathy” OR “nerve inflammation” OR “neuropathy”) | No |
| AND | ("Sciatic Neuropathy"[Mesh] OR "Sciatica"[Mesh] OR "sciatica" OR "Sciatic Nerve"[Mesh] OR "sciatic nerve") | No |
| AND | ("Physical Conditioning, Animal"[Mesh] OR “Physical therapy modalities”[Mesh] OR “physical therap*” OR “physiotherap*” OR “Exercise”[Mesh] OR “exercise therapy” OR “exercise*” OR “sham exercise” OR “run*” OR “swim*” OR “walk*” OR “Locomotion”[Mesh] OR “strength*” OR “endurance” OR “resistance” OR “physical conditioning” OR “motor activity” OR “aerobic*” OR “isometric*” OR “isotonic*” OR “isokinetic*” OR “resistance” OR “train*” OR “treadmill” OR “vibration”) | No |

**Scopus Search Formula ELSEVIER**

Original search: 1513

Updated search: 1694

| **Search number** | **Query** | **Filters** |
| --- | --- | --- |
| **#1** | (TITLE-ABS-KEY ( "Models, Animal" OR "animal*" OR "Mice" OR "Rats" OR "transgenic mice" OR "rodent" ) ) | No |
| AND | (TITLE-ABS-KEY ( "Pain" OR "Peripheral Nervous System Diseases" OR "Neuropathic pain" OR "Nerve pain" OR "Neuralgia" OR "nerve block method*" OR "nerve crush" OR "nerve constriction" OR "nerve crush" OR "nerve cut" OR "nerve injury" OR "nerve transection" OR "nerve ligation" OR "chronic constriction injury" OR "peripheral neuropathy" OR "nerve inflammation" OR "neuropathy" ) ) | No |
| AND | (TITLE-ABS-KEY ( "Sciatic Neuropathy" OR "Sciatica" OR "sciatic nerve" ) ) | No |
| AND | ( TITLE-ABS-KEY ( "Physical Conditioning, Animal" OR "Physical therapy modalities" OR "physical therap*" OR "physiotherap" OR "exercise therapy" OR "exercise*" OR "sham exercise" OR "run*" OR "swim*" OR "wal*k" OR "Locomotion" OR "strength*" OR "endurance" OR "resistance" OR "physical conditioning" OR "motor activity" OR "aerobic*" OR "isometric*" OR "isotonic*" OR "isokinetic*" OR "resistance" OR "train" OR "treadmill" OR "vibration" ) ) | No |

**WoS Search Formula ELSEVIER**

Original search: 861

Updated search: 861

| Search number | Query | Filters |
| --- | --- | --- |
| #1 | TS=("Models, Animal” OR “animal*” OR "Mice" OR "Rats" OR “transgenic mice” OR “rodent”) 2.934029 | No |
| #2 | TS=("Pain" OR "Peripheral Nervous System Diseases" OR "Neuropathic pain" OR ?Nerve pain? OR ?Neuralgia? OR ?nerve block method*? OR ?nerve crush? OR ?nerve constriction? OR ?nerve crush? OR ?nerve cut? OR ?nerve injury? OR ?nerve transection? OR ?nerve ligation? OR ?chronic constriction injury? OR ?peripheral neuropathy? OR ?nerve inflammation? OR ?neuropathy?) 790.561 | No |
| #3 | TS=("Sciatic Neuropathy" OR "sciatica" OR "sciatic nerve") 27.118 | No |
| #4 | TS=("Physical Conditioning, Animal" OR ?Physical therapy modalities? OR ?physical therap*? OR ?physiotherap*? OR ?Exercise? OR ?exercise therapy? OR ?exercise*? OR ?sham exercise? OR ?run*? OR ?swim*? OR ?walk*? OR ?Locomotion? OR ?strength*? OR ?endurance? OR ?resistance? OR ?physical conditioning? OR ?motor activity? OR ?aerobic*? OR ?isometric*? OR ?isotonic*? OR ?isokinetic*? OR ?resistance? OR ?train*? OR ?treadmill? OR ?vibration?) 5.812.530 | No |
| #5 | AND #3 AND #2 AND #1 861 | No |

**EMBASE**

Original search: 2236

Updated search: 2642

| Search number | Query | Filters |
| --- | --- | --- |
| #1 | (Animal model’ OR animal OR mouse OR mice OR rat* OR ‘transgenic mice’ OR ‘transgenic mouse’ OR rodent*) | No |
| #2 | (Pain OR ‘Peripheral Nervous System Diseases’ OR ‘Neuropathic pain’ OR ‘Nerve pain’ OR ‘Neuralgia’ OR ‘nerve block method*’ OR ‘nerve crush’ OR ‘nerve constriction’ OR ‘nerve cut’ OR ‘nerve injury’ OR ‘nerve transection’ OR ‘nerve ligation’ OR ‘chronic constriction injury’ OR ‘peripheral neuropathy’ OR ‘nerve inflammation’ OR ‘neuropathy’) | No |
| #3 | ‘Sciatic Neuropathy’ OR ‘Sciatica’ OR ‘Sciatic Nerve’ | No |
| #4 | Physical Conditioning, Animal’ OR ‘Physical therapy modalities’ OR ‘physical therap*’ OR ‘physiotherap*’ OR ‘exercise therapy’ OR ‘exercise*’ OR ‘sham exercise’ OR ‘run*’ OR ‘swim*’ OR ‘walk*’ OR ‘Locomotion’ OR ‘strength*’ OR ‘endurance’ OR ‘resistance’ OR ‘physical conditioning’ OR ‘motor activity’ OR ‘aerobic*’ OR ‘isometric*’ OR ‘isotonic*’ OR ‘isokinetic*’ OR ‘train*’ OR ‘treadmill’ OR ‘vibration’ | No |
| #5 | 1 AND 2 AND 3 AND 4 | No |

**Cochrane Library Search Formula**

Original search: 5

Updated search:11

| **Search number** | **Query** | **Filters** |
| --- | --- | --- |
| #1 | MeSH descriptor: [Models, Animal] explode all trees 435 | No |
| #2 | (“animal*”):ti,ab,kw 17979 | No |
| #3 | MeSH descriptor: [Mice] explode all trees 1014 | No |
| #4 | MeSH descriptor: [Rats] explode all trees 983 | No |
| #5 | (transgenicmice):ti,ab,kw 201 | No |
| #6 | (rodent):ti,ab,kw 683 | No |
| #7 | MeSH descriptor: [Pain] explode all trees 49844 | No |
| #8 | (Pain):ti,ab,kw 184032 | No |
| #9 | #9 MeSH descriptor: [Peripheral Nervous System Diseases] explode all trees 5573 | No |
| #10 | (neuropathic pain):ti,ab,kw 3786 | No |
| #11 | (nerve pain):ti,ab,kw 14101 | No |
| #12 | MeSH descriptor: [Neuralgia] explode all trees 1693 | No |
| #13 | (nerve block method):ti,ab,kw 3277 | No |
| #14 | (nerve crush):ti,ab,kw 36 | No |
| #15 | (nerve constriction):ti,ab,kw 0 | No |
| #16 | (nerve cut):ti,ab,kw 195 | No |
| #17 | (nerve injury):ti,ab,kw 2761 | No |
| #18 | (nerve transection):ti,ab,kw 68 | No |
| #19 | (nerve ligation):ti,ab,kw 105 | No |
| #20 | (chronic constriction injury):ti,ab,kw 20 | No |
| #21 | (peripheral neuropathy):ti,ab,kw 5506 | No |
| #22 | (nerve inflammation):ti,ab,kw 821 | No |
| #23 | (neuropathy):ti,ab,kw 10285 | No |
| #24 | MeSH descriptor: [Sciatic Neuropathy] explode all trees 326 | No |
| #25 | MeSH descriptor: [Sciatica] explode all trees 311 | No |
| #26 | (sciatica):ti,ab,kw 940 | No |
| #27 | MeSH descriptor: [Sciatic Nerve] explode all trees 580 | No |
| #28 | (sciatic nerve):ti,ab,kw 1027 | No |
| #29 | MeSH descriptor: [Physical Conditioning, Animal] explode all trees 17 | No |
| #30 | MeSH descriptor: [Physical Therapy Modalities] explode all trees 25881 | No |
| #31 | ("physical therapy"):ti,ab,kw 9693 | No |
| #32 | (physiotherap*):ti,ab,kw 16159 | No |
| #33 | MeSH descriptor: [Exercise] explode all trees 24831 | No |
| #34 | (exercise therapy):ti,ab,kw 40738 | No |
| #35 | (exercise*):ti,ab,kw 105617 | No |
| #36 | (sham exercise):ti,ab,kw 1513 | No |
| #37 | (run*):ti,ab,kw 22265 | No |
| #38 | (swim*):ti,ab,kw 1341 | No |
| #39 | (walk*):ti,ab,kw 31635 | No |
| #40 | MeSH descriptor: [Locomotion] explode all trees 8243 | No |
| #41 | (strenght*):ti,ab,kw 162 | No |
| #42 | (endurance):ti,ab,kw 10786 | No |
| #43 | (resistance):ti,ab,kw 59042 | No |
| #44 | (physical conditioning):ti,ab,kw 1332 | No |
| #45 | (motor activity):ti,ab,kw 10269 | No |
| #46 | (aerobic*):ti,ab,kw 15826 | No |
| #47 | (isometric*):ti,ab,kw 5829 | No |
| #48 | (isotonic*):ti,ab,kw 3243 | No |
| #49 | (isokinetic*):ti,ab,kw 2694 | No |
| #50 | (resistance):ti,ab,kw 59042 | No |
| #51 | (train*):ti,ab,kw 110042 | No |
| #52 | (treadmill):ti,ab,kw 8412 | No |
| #53 | (vibration):ti,ab,kw 3239 | No |
| #54 | {OR #1-#6} 19974 | No |
| #55 | {OR#7-#23} 201499 | No |
| #56 | {OR #24-#28} 2238 | No |
| #57 | {OR #29-#53} 275955 | No |
| #58 | {AND #54-#57} 5 | No |
