# Supplementary material for: Effect of Type and Dose of Exercise on Neuropathic Pain after Experimental Sciatic Nerve Injury: a Preclinical Systematic Review and Meta-analysis
Source: J Pain. Author manuscript; Available in PMC 2026 Jun 17. (PMC7619194; doi:10.1016/j.jpain.2023.01.011)
Supplement: Supplementary Table 2 [file EMS213962-supplement-Supplementary_Table_2.docx]

**Supplementary Table 2. Summary of CAMARADES tool outcomes**

*Item not analysed.
