# Supplementary material for: Effect of Type and Dose of Exercise on Neuropathic Pain after Experimental Sciatic Nerve Injury: a Preclinical Systematic Review and Meta-analysis
Source: J Pain. Author manuscript; Available in PMC 2026 Jun 17. (PMC7619194; doi:10.1016/j.jpain.2023.01.011)
Supplement: Supplementary Table 3 [file EMS213962-supplement-Supplementary_Table_3.docx]

**Supplementary Table 3: Types and doses of exercise used in the included studies**

| Study | Intervention | Intensity | Duration _total (min)_ | Duration _per day (min)_ |
| --- | --- | --- | --- | --- |
| Almeida, 2015 | Swim | Low | 950 | 27.5 |
| Fazard, 2017 | Swim | Low | 900 | 33.9 |
| Gaffuri,2011 | Swim | Low  High | 150 | 10.7 |
| Guo, 2021 | Swim | Low | 930 | 33.21 |
| Bobinski, 2011 | Run | Low | 300 | 21.4 |
| Bobinski, 2015 | Run | Low | 300 | 21.4 |
| Bobinski, 2018 | Run | Low | 300 | 21.4 |
| Chen, 2012 | Run | Low | Not clear | Not clear |
| Huang, 2017 | Run | High | 630 | 30 |
| Hung, 2016 | Run | High | 600 | 21.4 |
| Kami, 2016 | Run | Voluntary run | - | - |
| Kami, 2016b | Run | Low | 550 | 26.1 |
| Lopes, 2020 | Run | High | 160 | 20 |
| Martins, 2017 | Run | Low  High | 1200 | 21.4 |
| Safakhah, 2016 | Run | Medium | 300 | 21.4 |
| Safakhah, 2017 | Run | High | 550 | 26.1 |
| Sumizono, 2018 | Run | Low  Low | 450  750 | 12.8  21.4 |
| Taguchi,2015 | Run | Low | 300 | 60 |
| Tsai, 2017 | Run | Low  High | 630 | 30 |
| Antunes, 2016 | Other kind of exercise | Not enough data to classify | - | - |
| Gaffuri, 2011 | Other kind of exercise | Not enough data to classify | - | - |
| Malanotte, 2017 | Other kind of exercise | Not enough data to classify | - | - |
