# Supplementary material for: Effect of Type and Dose of Exercise on Neuropathic Pain after Experimental Sciatic Nerve Injury: a Preclinical Systematic Review and Meta-analysis
Source: J Pain. Author manuscript; Available in PMC 2026 Jun 17. (PMC7619194; doi:10.1016/j.jpain.2023.01.011)
Supplement: Supplementary Table 4 [file EMS213962-supplement-Supplementary_Table_4.docx]

**Supplementary Table 4: Biomarkers examined in the included studies**

| Reference | Anatomical level | Time point | Type of test | Biomarkers | Results |
| --- | --- | --- | --- | --- | --- |
| Almeida, 2015 | DRG | POD 42 and 70 | ELISA  Western blot | BDNF  GDNF  NGF  BDNF  GDNF  NGF  Iba-1  GFAP | Normal after 5 weeks of training  Decreased  Reduced, but not normalised  Normalised  Normalised |
| Ashour, 2017 | Blood  Nerve | POD 5 | Morphometric  PCR  ELISA | IL-6  IL-6R  STAT3 | Increased |
| Antunes, 2016 | Nerve  Leg | POD: 3, 7 | Western blot | BDNF  NGF  Edema assessment | No difference  Increased  No difference |
| Bobinsky, 2011 | Sciatic nerve  Spinal cord | POD 15 | ELISA | TNF-alfa  IL-1beta  IL-6R  IL-10  IL-1beta  IL-6R  IL-10 | Decreased  Decreased  No difference  No difference  Decreased  Decreased  No difference |
| Bobinsky, 2015 | Brainstem  Medullary raphe | POD 15 | ? | 5-HT  5-HIAA  5-HT1A  5-HT1B  5-HT2A  5-HT2C  5-HT3A  TNF-alfa  IL-1beta  SERT  Sert | Increased by exercise  Increased by exercise  No difference  Increased by exercise  Increased by exercise  Increased by exercise  No difference  Decreased by exercise  Decreased by exercise  Decreased by exercise  Decreased by exercise |
| Bobinsky, 2018 | Sciatic nerve  Spinal cord | POD 15 | Immunohistochemistry | IL-4  IL-1ra  IL-5  IL-6  IL-4  IL-1ra  IL-5  IL-6  BDNF  β-NGF  GFAP  Iba-1 | Increased by exercise  Increased by exercise  No difference  No difference  Increased by exercise  Increased by exercise  Increased by exercise  No difference  Decreased by exercise  Decreased by exercise  Decreased by exercise bilateral layers in the spinal cord I-II/ Ipsilateral III-VI  Decreased by exercise bilateral layers in the spinal cord I-II/ Ipsilateral III-VI |
| Bonetti, 2015 | Lumbar Spinal cord | POD 32 | Immunohistochemistry  Optical densitometry | Neurotrophin -3  Synaptophys | Increased by balance coordination training |
| Byun, 2005 | Sciatic nerve | POD 14 | RNA isolation  PCR | BDNF | Decreased |
| Chen, 2012 | Sciatic nerve | POD 21 | ELISA | Hsp72  TNF-alfa  IL-1beta | Increased by swimming  Increased by treadmill  Decreased by CCISE and treadmill  Decreased by swimming  Decreased by treadmill |
| Fazard, 2017 | Spinal cord | POD ?? | Western blot | Irisin protein  GAD65 | lower in CCI+swim  maintained CCI+swim |
| Huang, 2017 | Sciatic nerve | POD 14 and 28 | ELISA | TNF-a  IL-6  IL-10 | Decreased at both points  Decreased at both points  Increased at 14; no change at 28 |
| Guo, 2021 | Sciatic nerve | POD 28 | Hematoxyli-eosin staining | Schwann cells | Improved with exercise |
| Hung, 2016 | Spinal cord (L4 –L5) | POD 14 and 28 | ELISA | IL-6  IL-10  Iba1 IR | Decreased at both points  Increased POD 28  Decreased |
| Kami, 2016 | Ventral segmental area | POD 15 | Immunohistochemistry | TH enzyme  PCREB+ | Increased  Increased |
| Kami, 2016 b | Lumbar spinal cord | POD 7 | immunohistochemistry | GABA  GAD65/67 | Increased  Increased |
| Kami, 2020 | Amygdala | POD | Immunofluorescence analysis | FosB  GAD67  CGRP | Decreased  Decreased  Decreased |
| Korb 2010 | Spinal Cord  Magnus raphe nucleus  Dorsal raphe nucleus  Soleus muscle | POD 30 | Immunohistochemical  Optical Densitometry | Serotonin (5-HT) immunoreactivity (lumbosacral ventral horn)  Serotonin inmunoreactivity (superficial laminae of lumbosacral SC)  Serotonin inmunoreactivity (magnus raphe nucleus)  Serotonin inmunoreactivity (dorsal raphe nucleus)  Citrate synthase enzyme activity (soleus muscle) | Increased by training  No difference  No difference  No difference  Increased by training |
| Liao, 2017 | Spinal cord | POD 28 | Optical Densitometry | CGRP  Macrophage | Increased by Swimming+20% overload  No difference |
| Lopes, 2020 | Cerebral cortex  Brainstem  Spinal cord | POD 24 and 30 | ELISA | BDNF  Il-1b  Il-4  BDNF  Il-1b  Il-4  BDNF  Il-1b  Il-4 | Increased  Decreased  Increased  Decreased  Decreased  Increased  Increased  Increased  Increased |
| Martins, 2017 | Sciatic nerve  Triceps surae | POD 65 | ELISA | IL-1β  TNF-α  IL-4  IL-1Ra  IGF-1 | No difference  Muscle: Decreased by Exercise  Nerve: No difference  No difference  No difference  Nerve: Increased by exercise  Muscle: no difference |
| Safakhah, 2017 | Serum  Cerebrospinal fluid | POD 21 | Western Blot | MDA  Total antioxidant capacity  TNF-a | No change  Increased  Decreased |
| Seo, 2006 | Sciatic nerve | POD 14 | Immunohistochemistry  Cell culture  Western blot  PCR | Schwann cell  Schwann cell  Cdc 2  Cdc 2 | Increased  Increased  Increased  Increased |
| Seo, 2009 | Sciatic nerve  Dorsal root ganglia | POD 14 | Immunohistochemistry  Western blot | Schwann cell  GAP-43  ERK1/2 | Increased by low intensity treadmill training  Increased  Increased |
| Sumizono, 2018 | Lumbar spinal cord dorsal horn  Midbrain (PAG) | POD 38 | Immunohistochemistry | BDNF  MOR  GFAP  Iba 1  β-endorphin  Met-enkephalin | Decreased by high frequency exercise 5 weeks  Decreased by all exercise at 5 weeks  Decreased by all exercise at 5 weeks  Decreased by all exercise at 5 weeks  Increased by all exercise at 5 weeks  Increased by all exercise at 5 weeks |
| Taguchi, 2015 | Sciatic nerve | POD7 | Immunohistochemistry | CD68 M1  CD206 M2 | Decreased  Increased |
| Tsai, 2017 | Sciatic nerve | POD 26 | Lowry Protein Assay | IL-10  IL-6  TNF-a | Increased  Decreased  Decreased |
| Wang, 2016 | Tibia | POD 31 | Immunohistochemistry | Substance P | Decreased |

?: not reported; DRG: dorsal root ganglia; CCI: chronic constriction injury; POD: post operative day
