# Supplementary material for: Effect of Type and Dose of Exercise on Neuropathic Pain after Experimental Sciatic Nerve Injury: a Preclinical Systematic Review and Meta-analysis
Source: J Pain. Author manuscript; Available in PMC 2026 Jun 17. (PMC7619194; doi:10.1016/j.jpain.2023.01.011)
Supplement: Supplemental Figures [file EMS213962-supplement-Supplemental_Figures.pdf]

Figure legends:

Figure 1: Flow diagram of study selection and inclusion

Figure 2: Meta-analysis of the effect of exercise on mechanical hypersensitivity

Figure 3: Meta-analysis of the effect of exercise on heat hypersensitivity

Figure 4: Meta-analysis of the effect of exercise on cold hypersensitivity

Supplemental Figure 1: Assessment of publication bias. Visual inspection of the funnel plots does not suggest asymmetry. However, analysis for thermal hypersensitivity (B and C) are based on small numbers of studies. The dashed red line indicates the overall summary effect size. SMD: standardized mean difference.

Supplemental Figure 2: Meta-analysis of the effect of exercise on mechanical hypersensitivity according to species

Supplemental Figure 3: Meta-analysis on the effect of exercise on heat hypersensitivity according to species

Supplemental Figure 4: Meta-analysis on the effect of exercise on cold hypersensitivity according to species
